# The clinical value of optical genome mapping in the rapid characterization of *RB1* duplication and 15q23q24.2 triplication, for more appropriate prenatal genetic counselling

**DOI:** 10.1002/mgg3.2437

**Published:** 2024-04-08

**Authors:** Malek Bouassida, Denise Molina‐Gomes, Fairouz Koraichi, Bérénice Hervé, Morgane Lhuilier, Clémence Duvillier, Jessica Le Gall, Marion Gauthier‐Villars, Valérie Serazin, Thibaud Quibel, Rodolphe Dard, François Vialard

**Affiliations:** ^1^ Genetics Department CHI de Poissy‐St Germain en Laye Poissy France; ^2^ Obstetrics Department CHI de Poissy‐St Germain en Laye Poissy France; ^3^ Genetics Department Institut Curie Paris France; ^4^ RHuMA Team UMR‐BREED, UVSQ, INRAE, ENVA Montigny le Bretonneux France

**Keywords:** 15q23q24.2 triplication, optical genome mapping, prenatal diagnosis, *RB1* gene

## Abstract

**Background:**

Despite recent advances in prenatal genetic diagnosis, medical geneticists still face considerable difficulty in interpreting the clinical outcome of copy‐number‐variant duplications and defining the mechanisms underlying the formation of certain chromosomal rearrangements.

Optical genome mapping (OGM) is an emerging cytogenomic tool with proved ability to identify the full spectrum of cytogenetic aberrations.

**Methods:**

Here, we report on the use of OGM in a prenatal diagnosis setting. Detailed breakpoint mapping was used to determine the relative orientations of triplicated and duplicated segments in two unrelated foetuses harbouring chromosomal aberrations: a de novo 15q23q24.2 triplication and a paternally inherited 13q14.2 duplication that overlapped partially with the *RB1* gene.

**Results:**

OGM enabled us to suggest a plausible mechanism for the triplication and confirmed that the *RB1* duplication was direct oriented and *in tandem*. This enabled us to predict the pathogenic consequences, refine the prognosis and adapt the follow‐up and familial screening appropriately.

**Conclusion:**

Along with an increase in diagnostic rates, OGM can rapidly highlight genotype–phenotype correlations, improve genetic counselling and significantly influence prenatal management.

## Graphical abstract

The clinical significance of duplications and complex rearrangements is often difficult to ascertain. In this study, we used optical genome mapping in a prenatal diagnosis setting to characterize a 15q23q24.2 triplication and to determine the orientation of a duplication encompassing part of the *RB1* gene.

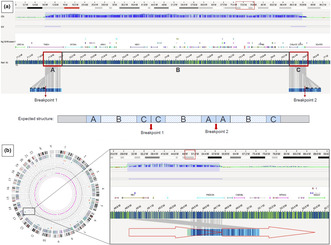

## 1 INTRODUCTION

Prenatal diagnostic testing (PDT) is now recommended for pregnancies with abnormal ultrasound findings and/or a high risk of aneuploidy. Due to their limitations, several techniques have to be combined for a reliable genetic diagnosis, which is generally costly and time‐consuming – especially when considering the time constraints of PDT (Chai et al., 2019).

Recently, optical genome mapping (OGM) has emerged as a next‐generation cytogenomic tool for the comprehensive analysis of structural variants (SVs) (Sahajpal et al., n.d.; Goumy et al., 2023; Mantere et al., 2021; Sahajpal et al., 2021). OGM provides a high level of genome coverage, ultra‐high resolution, a low false call rate and ease of analysis (Sahajpal et al., n.d.; Dremsek et al., 2021; Goumy et al., 2023; Mantere et al., 2021; Sahajpal et al., 2021).

Although disease‐causing deletions obviously lead to haploinsufficiency, the clinical significance of most duplications is difficult to establish; the phenotypes cannot always be explained by triplosensitivity. Partial duplications might lead to the disruption, dysregulation or fusion of genes spanning the breakpoints (Newman et al., 2015). Unlike inverted and inserted duplications, direct tandem duplications maintain a copy of the intact gene at the edges of the duplication (Newman et al., 2015). An illustrative example of this problem reported here is the duplication of the *RB1* tumour‐suppressor gene (OMIM#180200). Germline mutations in *RB1* confer a predisposition to retinoblastoma (Lan et al., 2020). The clinical impact of *RB1* duplications requires further assessment because of the importance of early diagnosis for the patients and their families.

In other cases, we lack a thorough understanding of the molecular mechanisms underlying complex chromosomal aberrations. Detailed breakpoint mapping is required to resolve the local genomic architecture. In the present study, we reasoned that elucidating the genomic structure of a 15q23q24.2 triplication might help to propose a mechanism for this rare genomic event resulting in a deleterious phenotype.

Hence, the objectives of the present study were to assess the ability of OGM to characterize complex constitutional chromosomal aberrations and to determine the orientation of duplications that are difficult to interpret in the context of PDT.

## 2 MATERIALS AND METHODS

### 2.1 Ethical compliance

In compliance with the Declaration of Helsinki, informed written consent for genetic study was obtained from participated individuals.

### 2.2 Samples and clinical data

#### 2.2.1 Case 1

A 29‐year‐old woman (gravida 2, para 1) was referred at 31 weeks of gestation (WG) for the genetic diagnosis of a triventricular hydrocephalus in a male foetus. There was no family history of congenital malformations. The second‐trimester ultrasound scan at 22 WG found that the lateral ventricles of the brain were 10.5 and 9.5 mm wide, and an ultrasound scan at 25 WG revealed that the third ventricle was enlarged. A further ultrasound scan at 29 WG revealed the presence of bilateral anechoic cysts near germinal zones, together with triventricular hydrocephalus. The findings for other internal organs were unremarkable.

#### 2.2.2 Case 2

A 34‐year‐old woman (gravida 3, para 2) with a dichorionic, diamniotic twin pregnancy underwent amniocentesis at 19 WG. The indication for amniocentesis was abnormal ultrasonographic findings at 18 WG, which were suggestive of pulmonary atresia and a ventricular heart defect in twin B. There was no family history of congenital malformations.

### 2.3 Standard molecular‐cytogenetic analyses

For both cases, standard G‐banding karyotyping was initially carried out on metaphases prepared from an amniotic cell culture. Between 350 and 400 bands were resolved.

For chromosomal microarray analysis (CMA), we used an Agilent Human SurePrint G3 Human CGH array Kit 8x60k oligonucleotide microarray (Agilent Technologies, Santa‐Clara, CA, USA) to examine DNA extracted from amniotic fluid or peripheral blood. The DNA was labelled and hybridized according to the manufacturer's instructions, and the arrays were scanned with an Agilent DNA Microarray Scanner. The array copy number data were analysed using Agilent Cytogenomics software, with a resolution of 1000 kb. Map positions refer to the GRCh37/hg19 Genome Assembly, and the nomenclature comes from the International System for Human Cytogenomic Nomenclature (2020) (McGowan‐Jordan et al., 2020).

To confirm the CMA results, a fluorescence in situ hybridization analysis was based on the fluorogen‐labelled probes RP11‐243B22 (15q24.1) and D15S936 (telomere 15q). The segregation analysis of polymorphic loci in the proband and the parents was based on the microsatellites D15S165, D15S153 (15q22.31), D15S113 (15q12) and D15S11 (15q11.2), spanning the long arm of chromosome 15.

### 2.4 Exome sequencing

The second case underwent trio‐based exome sequencing, consisting of the affected proband and the unaffected parents. To this end, 50 ng of fragmented DNA was amplified and enriched with Twist Human Exome Kit (Twist Bioscience, San Francisco, CA, USA). Sequencing was performed on Illumina NextSeq 2000 platform (Illumina, San Diego, CA, USA) in paired‐end mode (2 × 150bp). Raw data (bcl files) were converted to FASTQ files using DRAGEN software (Illumina, San Diego, CA, USA). Sequences were analysed following the Broad Institute's GATK (https://software.broadinstitute.org/gatk/) best practice guidelines. The reads were mapped to the Human Genome Build GRCh37/UCSC hg19. Raw data were analysed using the SeqOne genomics interpretation platform. Variants were annotated according to the Human Genome Variation Society (HGVS) recommendations (den Dunnen et al., 2016), and classified according to the American College of Medical Genetics and Genomics (ACMG) guidelines (Richards et al., 2015; Riggs et al., 2020).

### 2.5 Optical genome mapping

#### 2.5.1 Extraction and labelling of UHMW gDNA and chip loading

For prenatal samples or peripheral blood samples, ultra‐high molecular weight (UHMW) genomic DNA (gDNA) was isolated from cultured amniocytes according to the manufacturer's instructions (Bionano Prep SP Blood and Cell DNA Isolation Kit; Bionano Genomics, San Diego, CA, USA).

Briefly, a lysis, binding, washing, and elution procedure is used to release UHMW gDNA. The gDNA was then labelled at specific sequence motifs with a direct label and stain kit (Bionano Prep DLS Labelling Kit; Bionano Genomics) according to the manufacturer's protocols. For that purpose, direct label enzyme (DLE‐1) with a green fluorophore was used to label 750 ng of gDNA at the CTTAAG sequences.

The labelled UHMW gDNA molecules were loaded onto a Saphyr nanochannel chip for linearization and imaging (Bionano Genomics).

#### 2.5.2 De novo assembly and data analysis

Bionano Solve™ software was used for de novo assembly of captured long DNA molecules and reconstruction of a whole‐genome map. The data were analyzed via two pipelines included in the Bionano Access software package: a copy number variation (CNV) coverage‐based pipeline for the detection of large, unbalanced aberrations, and an SV pipeline based on the comparison of labelling patterns and alignment of the sample assembly with the GRCh37 reference genome map. This comparison can identify germline SVs such as insertions, duplications, deletions, inversions, and translocations.

The variants were filtered according to defined guidelines, including the manufacturer's recommended confidence values for SV/CNV calls (to filter out false positives), the hg19 DLE‐1 SV mask filter (to hide highly repetitive sequences), a Bionano control database of more than 300 apparently healthy individuals (to exclude common SVs and potential artefacts), and a filter for variants that overlap with morbid genes (precision: 12 kbp for SVs and 500 kbp for CNVs). A minimum size of 500 kb was selected for CNVs called by the read depth pipeline.

Subsequently, SVs and CNVs detected by OGM were compared with clinically relevant aberrations previously identified in CMA.

In compliance with the Declaration of Helsinki, informed written consent for genetic study was obtained from participants or their guardians.

## 3 RESULTS

### 3.1 Case 1

CMA of the proband revealed a de novo 4.8 Mb triplication at 15q23q24.2 (arr[GRCh37] 15q23q24.2(71738623_76597469)x4) that included 18 disease‐causing genes (according to the Online Mendelian Inheritance in Man (OMIM) database): *THSD4, NR2E3, MYO9A, HEXA, BBS4, HCN4, REC114, LOXL1, PML, STRA6, CYP11A1, SEMA7A, EDC3, MPI, COX5A, MAN2C1, SIN3A*, and *ETFA* (Figure S1). None of these genes has been linked to a triplosensitive phenotype. *SIN3A* variants have been associated with cerebral abnormalities, while *SEMA7A* and *CPLX3* genes might have an important role in brain development (Balasubramanian et al., 2021; El‐Hattab et al., 2010). No other significant chromosomal imbalances (i.e. that might have explained the abnormal brain structure) were detected. Segregation analysis showed biparental contribution to microsatellite polymorphisms within 15q. Only the polymorphism D15S165 located at 15q13.3 was informative and did not map within the rearrangement locus. The parents opted to terminate the pregnancy.

**FIGURE 1:**
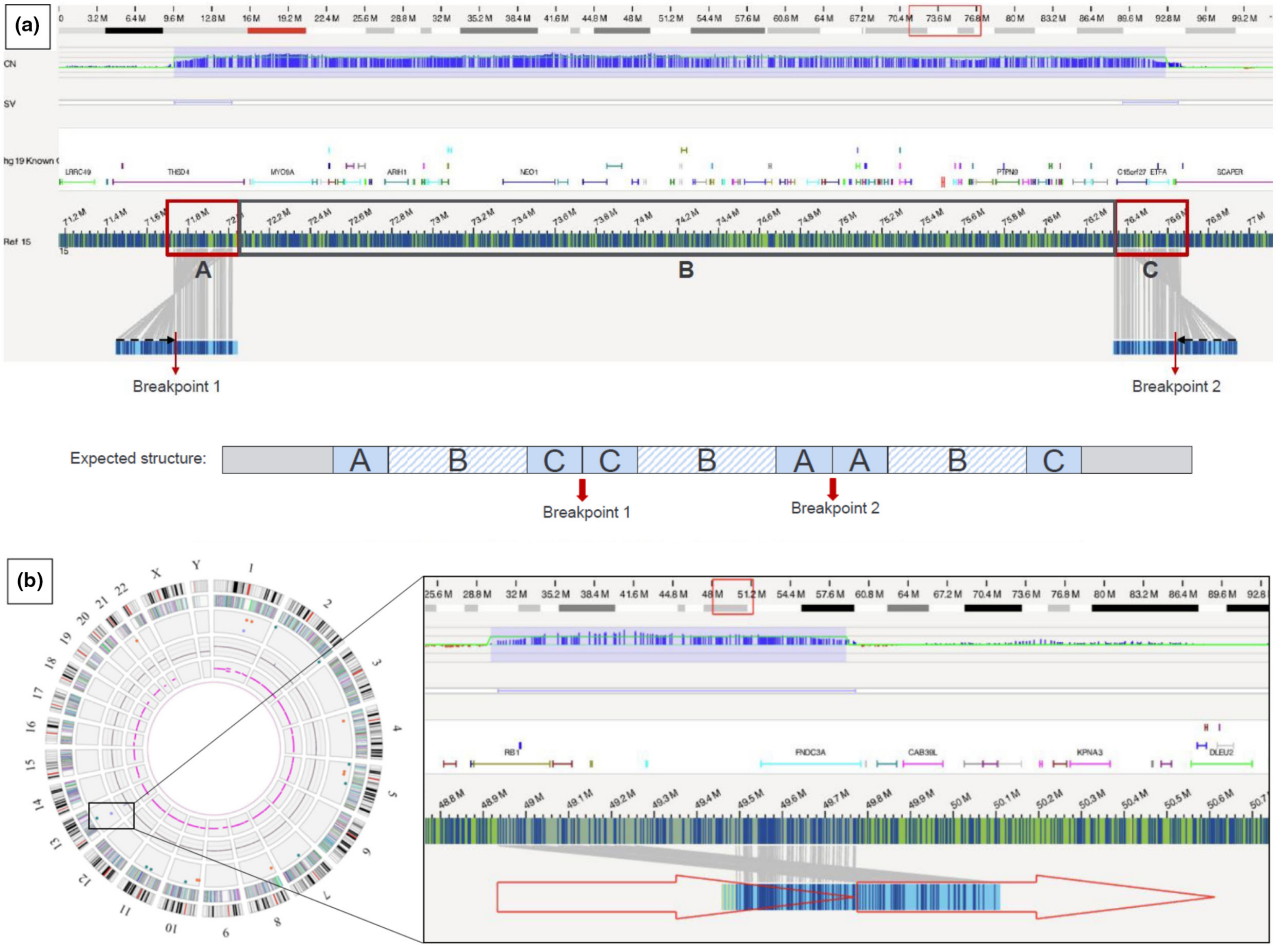
Schematic representation of the intrachromosomal triplication (a) and the 13q14.2 duplication (b) identified by OGM. (a): The SV track shows small maps that span each breakpoint in the middle segment represented by a “fold‐back” inversion of labels (black arrows), which indicates a sense‐inverted‐sense triplication. (b): Left panel: in the Circos plot, the duplication is shown as a blue line in the copy number track (highlighted with a blue box). Right panel: The SV pipeline calls for a duplication‐split with double alignment with the reference, indicating a tandem duplication.

OGM of the proband's sample revealed that the middle segment of the triplication was inverted, relative to the other segments (Figure 1a). On this basis, we hypothesized that the triplication arose from a meiotic error. This mechanism has a low risk of recurrence, which prompted us to suggest to the couple that the deleterious rearrangement was probably sporadic but that PDT of further pregnancies would be possible.

### 3.2 Case 2

CMA highlighted a 0.82 Mb duplication in the 13q14.2 region (arr[GRCh37]13q14.2(48950869_49772260)x3) that involved two disease‐causing genes (according to the OMIM database): all of *LPAR6* and exons 13 to 27 of *RB1*. Parental CMA revealed that the duplication is inherited from the father, who *exhibits a normal phenotype*. Exome sequencing confirmed the presence of the duplication but did not reveal the orientation of the duplicated segments. According to the guidelines issued by the AchroPuce network, we interpreted the duplication as a variant of uncertain significance.

OGM of the father's DNA revealed a tandem intergenic duplication with directly oriented segments (Figure 1b). These results prompted us to reconsider our interpretation of the variant's significance and classify it as likely benign. We informed the family about the clinical significance of this incidentally discovered duplication and reassured them with regard to the prognosis. In light of these results, the pregnancy was continued. The child was screened by an ophthalmologist at birth and regularly thereafter. No retinoblastoma was observed more than a year after birth. The father's fundus did not show any signs of retinoblastoma.

## 4 DISCUSSION AND CONCLUSION

The clinical interpretation of duplications and complex rearrangements identified by CMA is challenging for medical geneticists. In the present study, we used OGM to clarify the clinical significance of a duplication encompassing part of the *RB1* gene and to characterize the genomic structure of a 15q23q24.2 triplication.

To the best of our knowledge, our study is the first to have detected a triplication within the 15q23q24 locus. Interstitial triplications generally lead to a phenotype that is consistent with (but more severe than) duplication of the same locus (Soler‐Alfonso et al., 2014). An appropriate clinical interpretation requires further assessment of the genomic CNV content.

Interestingly, the 15q23q24 region contains five low copy repeats (LCRs): LCRs A (BP4), B (BP1), C, D (BP2) and E (BP3) (Liu et al., 2020; Mefford et al., 2012). The occurrence of several recurrent 15q24 microdeletions (Mefford et al., 2012) led to the identification of a new clinically recognizable syndrome defined as Witteveen‐Kolk syndrome (WITKOS; OMIM:613406; ORPHA:94065) and *SIN3A* as the causative gene. The main reported features are global developmental delay, mild‐to‐moderate intellectual disability, and hypotonia in more than 75% of cases, digital anomalies in 50–75% of cases, and cerebral malformations (including enlarged ventricles) in 25–50% of cases (Liu et al., 2020; Magoulas & El‐Hattab, 2012; Mefford et al., 2012). The majority of deletions are between 1.7 to 6.1 Mb in size, with breakpoints located within the five LCR clusters. The first minimal critical region (MCR) to be defined spans 1.2 Mb between LCR‐B and LCR‐C (Magoulas & El‐Hattab, 2012). This region has been linked to developmental delay and congenital malformations (Magoulas & El‐Hattab, 2012; Mefford et al., 2012). The proband's triplication included the MCR; however; the proximal breakpoint was 1.1 Mb centromeric of LCR‐A, and the distal breakpoint was 0.48 Mb telomeric of LCR‐D.

To date, twelve 15q24 duplications have been reported in patients sharing several clinical features with the microdeletion carriers (Liu et al., 2020). Most of the duplications' breakpoints lie within LCRs and include the same MCR as for reciprocal microdeletions (Liu et al., 2020). Due to the relatively small number of microduplications described to date, the clinical phenotypes have not yet been well delineated. Indeed, the size and gene content of the triplicated region and the phenotype observed in overlapping imbalances strongly suggest that the triplication is clinically significant.

OGM enabled us to rapidly infer the genomic architecture of the triplication, with inversion of the middle segment. We suggest that this chromosomal rearrangement results from two crossovers within the inversion loop, with a U‐type exchange at the pachytene stage of the first meiotic prophase (Figure S2) (Vialard et al., 2003). This finding comes along with the mechanisms that have been proposed for triplications that are not embedded within duplicated segments and that usually involve flanking LCRs (such as the triplications identified in the Xp22.31 region including the *STS* gene; Liu et al., 2011) (Weckselblatt & Rudd, 2015). However, it is worth noting that replication‐based mechanisms, such as Break‐Induced Replication (BIR), Fork Stalling and Template Switching (FoSTeS) and/or Microhomology‐Mediated Break Induced Replication (MMBIR), are thought to explain the formation of inverted triplications embedded within duplicated segments (DUP‐TRP/INV‐DUP pattern) at the *MECP2* and *PLP1* loci (Carvalho et al., 2011). Similarly, Nicolle et al. used OGM to characterize the mechanism of a 16p13.11p11.2 triplication (Nicolle et al., 2022). RNA studies were not justified as none of the genes (*THSD4*, *ETFA*) lying within the triplication breakpoints are predicted to form fusion transcripts. Although the triplication was de novo, the inverted model allowed to predict the genetic recurrence risk to be relatively low as it arose from a sporadic meiotic error. We believe that our case provides information on possible mechanisms underlying intrachromosomal triplications. However, additional patients are required to address genotype–phenotype correlations.

Our second case report concerned a 0.82 Mb microduplication that included part of the *RB1* gene and was inherited from an asymptomatic father. Heterozygous germline pathogenic variants in *RB1* confer autosomal‐dominant susceptibility to retinoblastoma (Lan et al., 2020). Patients carrying these variants should therefore receive personalized clinical follow‐up. Although large duplications/deletions account for 16% of *RB1* deficiencies (Lan et al., 2020), partial duplications involving this gene are rarely described in the literature. Although retinoblastoma is generally associated with high penetrance, some familial retinoblastomas display low penetrance and variable expressivity (Alekseeva et al., 2021); hence, phenotype prediction and prenatal genetic counselling are very challenging.

Our OGM analysis revealed that the 13q14.2 duplication was *in tandem* and direct oriented. These findings are in line with a detailed breakpoints mapping study of 184 germline duplications, most of which were *in tandem* and directly oriented (Newman et al., 2015). Based on the current evidence, it is reasonable to conclude that a functional copy of *RB1* is maintained and that this small duplication is unlikely to cause disease. For example, a tandem duplication of *BRCA1* exons 1–19 through to *DHX8* exon 2 was considered as probably a benign variant in a recent study (Du et al., 2018). Intergenic duplications can also fuse genes if the latter have the same orientation and if the reading frame is maintained (Newman et al., 2015). Consequently, messenger RNA and protein expression studies might be justified if the tested patient has a personal or family history of retinoblastoma. It should be noted that in the present case, the father's fundus showed no signs of retinoblastoma.

These two cases highlight the clinical value of OGM for the genomic characterization of CNVs of clinical relevance. As a fast, accurate single assay, OGM has the potential to replace current technologies in a PDT setting, but studies on a larger scale are necessary to reinforce these findings.

## AUTHOR CONTRIBUTIONS

MB wrote the original draft; MB, DMG, FK, BH, CD, JLG, MGV, VS, TQ, RD and FV contributed to patients' evaluation, synthetizing and reviewing clinical data; ML participated in the experiments' conduction and data analysis; RD and FV reviewed and edited the manuscript. All authors have read and agreed to the published version of the manuscript.

## FUNDING INFORMATION

No funding was received for this study.

## CONFLICT OF INTEREST STATEMENT

The authors declare no conflict of interest.

## Supporting information


Figure S1.

Figure S2.

## Data Availability

The datasets generated for the current study are included in this article. Further inquiries can be directed to the corresponding authors.
