# Supplementary material for: The clinical value of optical genome mapping in the rapid characterization of RB1 duplication and 15q23q24.2 triplication, for more appropriate prenatal genetic counselling
Source: Mol Genet Genomic Med. 2024 Apr 8;12(4):e2437. doi: 10.1002/mgg3.2437 (PMC11000809; doi:10.1002/mgg3.2437)
Supplement: Supplementary file 1 — Figure S1. [file MGG3-12-e2437-s002.docx]

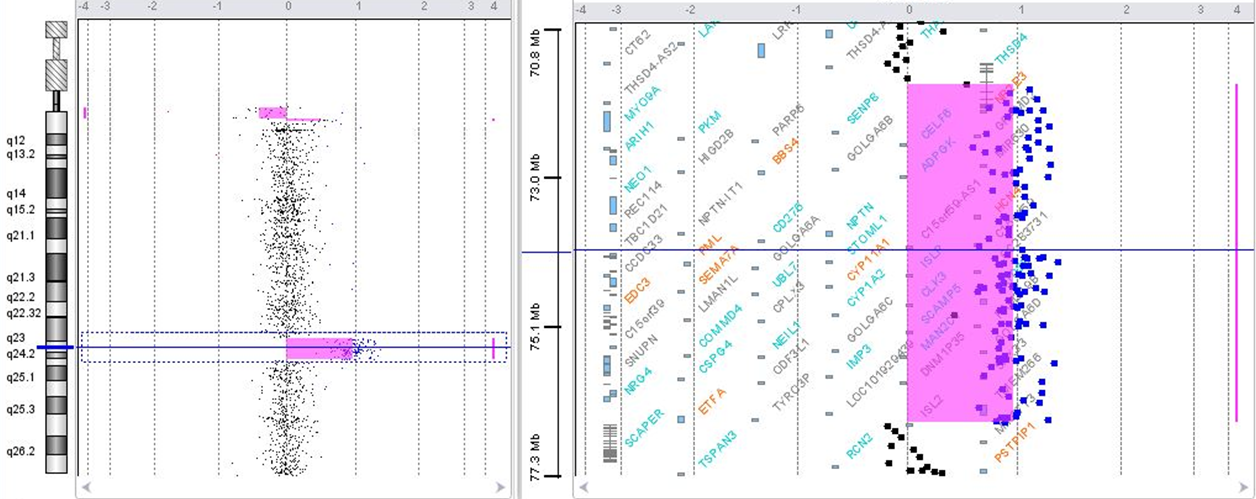
**Figure S1.** Comparative genomic hybridization analysis (SurePrint G3 CGH ISCA v2, 8 × 60K array (Agilent Technologies, Santa Clara, CA, USA) of DNA extracted from amniotic fluid, showing a 4.8 Mb triplication at 15q23q24.2 (71738623_76597469) that encompasses 18 morbid genes (according to OMIM).

In the right panel, the dots correspond to the oligonucleotide probes’ positions on chromosome 15. In the left panel, the CNV region is located at about 1.0 on the log 2 scale, which suggests the gain of two copies (i.e. triplication).
