# Supplementary material for: The clinical value of optical genome mapping in the rapid characterization of RB1 duplication and 15q23q24.2 triplication, for more appropriate prenatal genetic counselling
Source: Mol Genet Genomic Med. 2024 Apr 8;12(4):e2437. doi: 10.1002/mgg3.2437 (PMC11000809; doi:10.1002/mgg3.2437)
Supplement: Supplementary file 2 — Figure S2. [file MGG3-12-e2437-s001.docx]

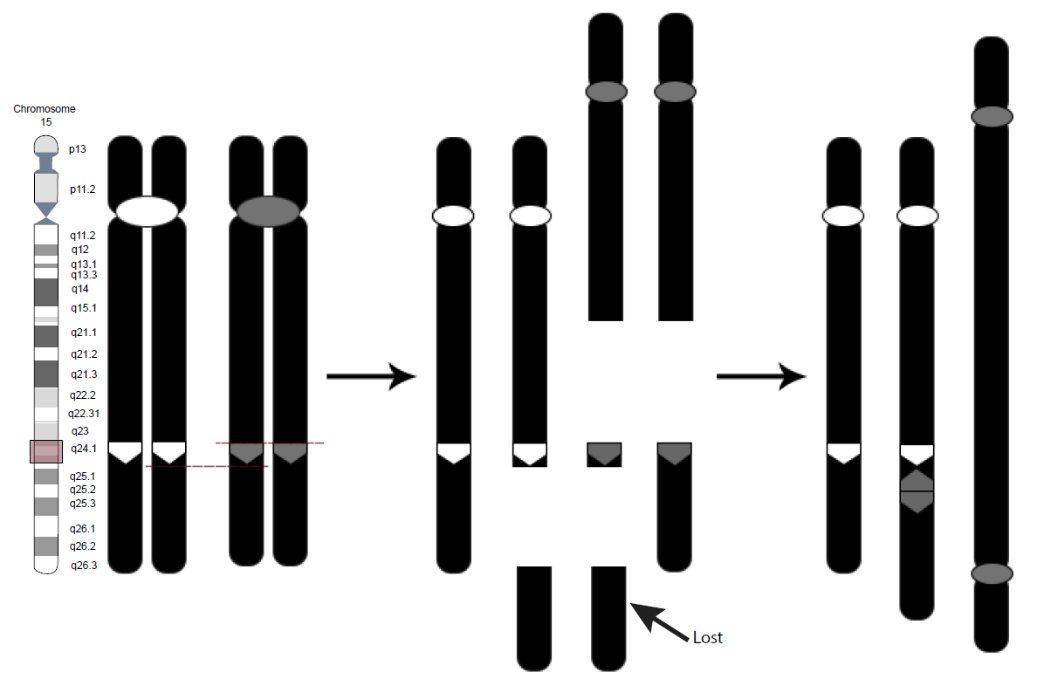


A

B

C

**Figure S2.** Schematic representation of the mechanism that might underlie the 15q23q24.2 intrachromosomal triplication identified in the foetus. The mechanism might involve a three-chromatid exchange mediated by two U-type exchanges and resulting from crossovers within the inversion loop at the pachytene stage of the first meiotic prophase. The first U-type exchange (A) involves a distal breakpoint region between homologous chromatids, while the second U-type exchange (B) occurs at the proximal breakpoint region between sister chromatids. We assume that the acentric derivative and the small inv dup(15) chromosome are lost. The resulting chromosome contains three sequentially arranged segments, indicating the presence of an interstitial triplication with an inverted repeat (C). Each repeat’s orientation is indicated by arrowheads.
